# Veterinary Department of Harvard University

**Published:** 1897-08

**Authors:** 


### COMMENCEMENT EXERCISES.

### VETERINARY DEPARTMENT OF HARVARD UNIVERSITY.

The graduating exercises of this department were held on June 30th, in conjunction with the closing exercises of the several departments of this time-honored institution. The conferring of the degrees took place in Saunders's Theatre, Cambridge, where the twenty successful candidates for the degree of M.D.V. were presented by Dean Lyman of the Veterinary Department, and their diplomas were presented by President Eliot of the University. The following are the names of the graduates: Frank Jerome Babbitt, Elmer Warren Babson, Clarence Edward Burchstead, Howard Montgomery Burgers, Cornelius Cronon, Frederick Thomas Dolan, Clement Arthur Hamblet, Edward Ambrose Madden, Arthur Winthrop May, William Augustine Nannery, Ellis Peterson, Jr., Charles Herbert Perry, Thomas Joseph Shinkwin, Frank Benjamin Stratton, Berton Amasa Thissell, Pell Fletcher Wallingford, Lewis Cummings Weeks, Daniel George White, William Tisdale White, and Alexander Eames Wight.

**The ladies' programme is complete.**

